# Mapping peak flowering phenology of rapeseed (*Brassica napus*) in North Dakota using Landsat 8 and Sentinel-2

**DOI:** 10.1038/s41598-026-55022-w

**Published:** 2026-06-01

**Authors:** Ehsan Rahimi, Chuleui Jung

**Affiliations:** 1 Agricultural Research Institute, Gyeongkuk National University, Andong, Republic of Korea; 2 Department of Plant Medical, Gyeongkuk National University, Andong, Republic of Korea

**Keywords:** Rapeseed flowering, Phenology, Agricultural monitoring, Flowering phenology, Vegetation indices, Pollination services, Ecology, Ecology, Environmental sciences, Plant sciences

## Abstract

Detecting the peak flowering time of rapeseed (*Brassica napus*) is essential for optimizing crop management, improving yield forecasts, and enhancing pollination services. This study aimed to map and analyze the spatial and temporal patterns of peak flowering in spring rapeseed across North Dakota using satellite remote sensing. We utilized multi-year optical imagery from Landsat 8 (2013–2024) and Sentinel-2 (2018–2024), applying the Normalized Difference Yellow Index (NDYI)—an index sensitive to yellow floral reflectance—to identify peak flowering dates. Our results indicate that peak flowering dates generally cluster around Julian day 200 (mid-July). Sentinel-2 provided more temporally consistent and spatially detailed estimates than Landsat, owing to its higher revisit frequency (5–7 days versus 16 days) and finer spatial resolution (10 m versus 30 m). On average, Sentinel-2 detected slightly later flowering dates (Julian days 203–230) compared to Landsat (196–213). Median peak flowering dates from Landsat ranged between Julian days 191 and 221, whereas Sentinel-2 medians were narrower and more closely aligned with the mid-July window, spanning Julian days 202 to 216. These differences underscore Sentinel-2’s superior capacity to capture flowering events precisely, while Landsat’s coarser temporal resolution occasionally resulted in earlier or delayed detections. Overall, the NDYI-based approach effectively captured flowering dynamics, revealing both inter-annual variability and field-level heterogeneity. These findings demonstrate the value of NDYI remote sensing for monitoring rapeseed phenology and suggest its broader applicability in agricultural and ecological contexts.

## Introduction

Rapeseed (*Brassica napus* Linnaeus.) (http://www.linneanonline.org/7723/), commonly known as rapeseed in North America, is a globally significant oilseed crop that plays a vital role in food, feed, and bioenergy production systems. Over the past four decades, global rapeseed production has grown substantially, positioning it as the second most cultivated oil crop worldwide^1,2^. Countries such as China, Canada, and members of the European Union are leading producers. For instance, China alone cultivates around 6.7 million hectares, contributing 12 million tons annually, which amounts to nearly 20% of the global rapeseed supply^3,4^. In Canada, rapeseed is planted on more than 20 million acres, comprising almost 30% of all cultivated cropland as of the 2016 Agricultural Census^5^.

Beyond its economic value, rapeseed is an ecologically relevant mass-flowering crop that offers temporally concentrated floral resources to pollinators. Rapeseed employs multiple pollination modes, but its floral traits—such as bright yellow, open flowers, high nectar production, and sticky pollen—are adapted for insect pollination^6^. These features attract a wide range of insect visitors, particularly honeybees, although they are often inefficient pollinators, as they fail to contact reproductive structures in the majority of visits^7^. More effective pollinators include solitary bees (*Andrena*, *Osmia* spp.), bumblebees, and hoverflies, with solitary bees depositing pollen in up to 71% of visits^8,9^. A diverse insect community improves pollination quality and enhances fruit set^10^. Pollinator dependency varies among cultivars, with some showing minimal yield response to insect visits^11–13^.

Overall, insect pollination contributes 10–40% of yield^14^, but even when combined with other factors like pest damage and soil fertility, it explains only about 20% of yield variability^15^. Importantly, flowering time within a single field is not spatially or temporally uniform. Microclimatic variation, soil heterogeneity, and management practices can cause asynchronous flowering patterns that affect how pollinators utilize fields and how effectively pollination is achieved^16^. Therefore, detecting within-field variation in flowering phenology has important applications for site-specific pollination management, pesticide scheduling, disease risk assessment, and yield forecasting^17,16^. Identifying the peak flowering date at the parcel level offers a key phenological marker that indicates when floral resource density is maximized, supporting better decisions in both agronomic and ecological management.

However, traditional methods to monitor rapeseed phenology—such as field surveys and manual scoring—are labor-intensive, subjective, and often spatially limited, making them infeasible for large-scale implementation^18^. These challenges underscore the need for efficient, scalable, and accurate techniques to assess flowering status and dynamics across entire agricultural regions. Optical remote sensing offers a promising solution to monitor flowering phenology, particularly using high-temporal and multispectral data from sensors like Sentinel-2. With 10–20 m spatial resolution and 5-day revisit time, Sentinel-2 allows for frequent, high-quality observations suitable for detecting phenological transitions in flowering crops^19^. The floral contribution to canopy reflectance during flowering is spectrally distinguishable, primarily through changes in visible bands, particularly the green (∼560 nm) and blue (∼490 nm) portions of the spectrum^20^. Yellow rapeseed flowers increase canopy reflectance in these bands, thereby modifying traditional vegetation indices. Commonly used vegetation indices such as NDVI (Normalized Difference Vegetation Index), which is based on NIR and red wavelengths, are often confounded during flowering periods due to increased red reflectance from yellow petals, leading to underestimated greenness^21^. NDVI, while linked to photosynthetic capacity and biomass, does not adequately capture non-green vegetation components like flowers.

To overcome these limitations, new spectral indices have been developed specifically to detect flowering signals, particularly for yellow-petaled crops like rapeseed. Among these, the Normalized Difference Yellowness Index (NDYI) has shown high sensitivity to flower abundance in rapeseed canopies. NDYI is defined as a normalized contrast between green and blue reflectance, emphasizing the spectral characteristics of yellow flowers, which reflect both green and red light due to carotenoid absorption at ~ 450 nm^22^. Field studies in Oregon demonstrated that NDYI correlates strongly (R² > 0.8) with the number of yellow flowers per unit area, outperforming NDVI during the flowering stage^20^. NDYI increases sharply as the canopy becomes dominated by yellow petals, peaks at maximum flowering, and declines as petals drop and the green vegetative canopy resumes dominance. Thus, NDYI offers a reliable phenological signal to determine the peak flowering period of rapeseed, with minimal interference from chlorophyll or vegetative structure.

Although NDYI has demonstrated strong potential for detecting flowering in rapeseed, it has yet to be applied at a nationwide scale, particularly within the United States, where rapeseed cultivation has been expanding in northern states such as North Dakota and Montana. In the United States, rapeseed cultivation—primarily in the form of spring rapeseed—is concentrated in northern states, especially North Dakota and Minnesota, which together account for the largest share of national production^21^. North Dakota alone dominates U.S. rapeseed cultivation, making it a key region for studying rapeseed phenology and management practices. Extensive yield trials conducted by North Dakota State University (NDSU) have evaluated a wide range of spring rapeseed cultivars, including both Roundup Ready and non-Roundup Ready varieties. These trials have shown comparable average yields—approximately 2342 pounds per acre for Roundup Ready types and 2,303 pounds per acre for non-Roundup Ready cultivars^23^.

Most prior studies have focused on localized field trials or regional applications in Europe and China, resulting in a significant geographic and methodological gap in our understanding of flowering phenology across broader landscapes. To address this gap, the present study aims to answer two key research questions: (1) Can NDYI derived from Landsat 8 and Sentinel-2 imagery be used to reliably estimate peak flowering dates of rapeseed at a large (state-wide) scale? and (2) What are the spatial and temporal patterns of rapeseed flowering phenology across North Dakota? To answer these questions, we implement a large-scale framework in which rapeseed fields are first identified and masked using the USDA Cropland Data Layer (CDL). NDYI time series are then computed for each field during the flowering season, and the date of maximum NDYI is extracted as a proxy for peak flowering. These dates are subsequently aggregated to generate spatially explicit maps of flowering phenology, allowing the analysis of regional variability and temporal dynamics.

## Methods

### Study area

**Fig. 1:**
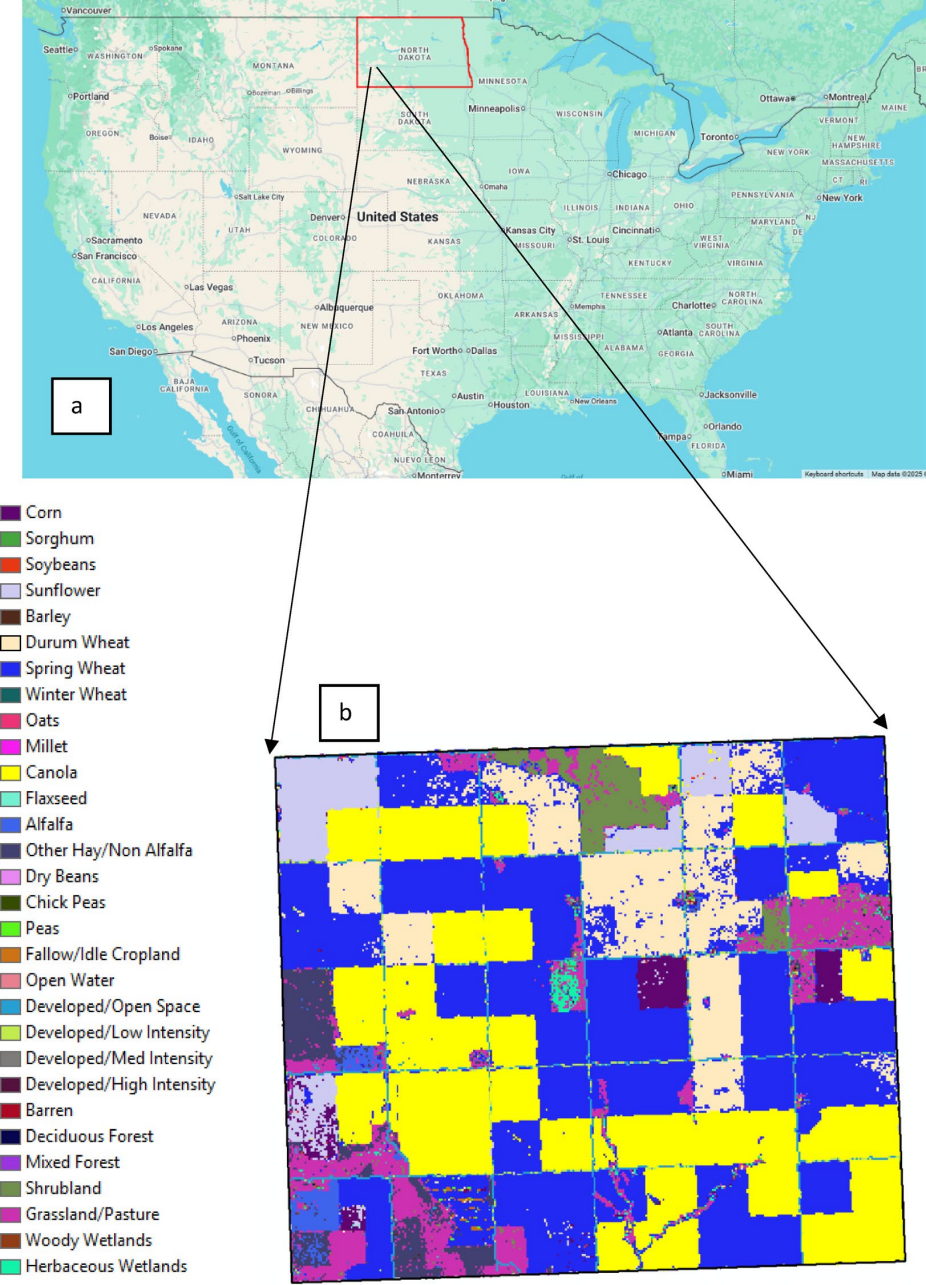
(**a**) Geographic location of North Dakota within the United States, highlighted as the study area for rapeseed flowering analysis. (**b**) A representative subset of cropland areas within North Dakota, illustrating the spatial distribution of agricultural fields used in the study.

In this study, we focus on rapeseed fields across North Dakota (Fig. 1), with a particular emphasis on detecting the peak flowering stage using remote sensing. In North Dakota, where spring rapeseed is typically sown between late April and early May, the peak flowering (BBCH 65–67) window usually occurs in mid-July for spring-sown varieties in North Dakota^18^. According to the BBCH phenological scale, rapeseed development progresses through distinct stages: emergence (00–09), leaf development (10–19), stem elongation (30–39), inflorescence emergence (50–59), flowering (60–69), and seed development to maturity (70–89)^24^.

During the growing season (April–August), mean monthly air temperatures typically increase from approximately 5–10 °C in April to 20–25 °C in July, followed by a gradual decline in August. Long-term climatological records indicate that canola flowering generally coincides with mid-summer thermal conditions, when temperature and solar radiation are at their peak. Precipitation during the same period is moderately variable, with average monthly totals ranging from approximately 50 to 100 mm, most of which occurs as convective rainfall events during late spring and summer. Interannual variability in both temperature and precipitation contributes to spatial and temporal heterogeneity in crop development and flowering onset.

Soils in North Dakota are predominantly derived from glacial till and lacustrine deposits and are classified mainly as Mollisols, which are highly fertile and support intensive rainfed agriculture. These soils are typically rich in organic matter, with good water-holding capacity, although soil moisture availability can vary considerably across the landscape due to differences in texture, topography, and precipitation distribution. Such variability in soil and climatic conditions contributes to differences in crop growth rates and phenological development across fields.

### Data

In this study, we employed multi-source satellite remote sensing data alongside land cover reference datasets to investigate the peak flowering period of spring rapeseed (*Brassica napus* L.) in North Dakota, USA. Two primary satellite datasets were used: Sentinel-2 and Landsat 8, each contributing differently to the analysis in terms of temporal coverage and spatial resolution. Sentinel-2 Surface Reflectance Harmonized imagery, available through Google Earth Engine, was used as the primary dataset for high-temporal-resolution analysis. This dataset includes atmospherically corrected surface reflectance from both Sentinel-2 A and Sentinel-2B satellites, with a spatial resolution of 10 m for visible bands and a revisit frequency of approximately 5 days.

To extend the analysis temporally, we incorporated Landsat 8 Collection 2 Level-2 surface reflectance data for the period 2013–2024, as well as Sentinel-2 data for 2018–2024. Landsat 8 provides a spatial resolution of 30 m and a revisit interval of 16 days, resulting in lower temporal density compared to Sentinel-2 but offering a longer historical record. On average, approximately 144 Landsat images per year and 1,077 Sentinel-2 scenes per year were processed for North Dakota. The integration of both datasets allowed us to assess the consistency of flowering detection across sensors with differing spatial and temporal characteristics.

All imagery was filtered using a ≤ 20% cloud-cover threshold at the scene level, followed by pixel-level cloud masking based on quality assurance (QA) bands (described in the Methods section). The 20% threshold represents a commonly used compromise in remote sensing studies, balancing data availability and quality. A stricter threshold (e.g., 10%) would substantially reduce the number of usable observations, particularly in regions with frequent cloud cover, potentially leading to temporal gaps during the critical flowering period. Conversely, a more lenient threshold could introduce excessive noise due to cloud contamination. In this study, residual cloud effects are further minimized through pixel-level masking and the use of a maximum-value compositing approach, which reduces the likelihood that cloud-contaminated pixels influence peak NDYI detection.

To spatially constrain the analysis to rapeseed fields, we used the USDA Cropland Data Layer (CDL) (USDA Cropland Data Layer (CDL), which provides annual crop-specific land cover classifications at 30 m resolution across the United States. Importantly, a separate CDL layer was used for each year of analysis, ensuring that interannual changes in crop distribution were explicitly accounted for. Rapeseed fields were identified using CDL class code 31, and a binary mask was applied to isolate these areas within North Dakota. This approach avoids potential inaccuracies associated with applying a single-year crop mask across multiple years. The CDL dataset is widely used and has demonstrated high classification accuracy for rapeseed. According to Lark et al.^25^, the CDL achieves a producer’s accuracy of 93.8% and a user’s accuracy of 89.1% for rapeseed classification, with Kappa coefficients of 0.938 and 0.891, respectively, based on extensive validation data. These performance metrics support the reliability of CDL as a reference dataset for crop-specific analyses.

### Estimating the peak of rapeseed flowering

In this study, we estimated the peak flowering date of spring rapeseed in North Dakota using time series of satellite-derived Normalized Difference Yellowness Index (NDYI) from Landsat 8 Surface Reflectance Tier 1 and Sentinel-2 Surface Reflectance imagery. The analysis was conducted through a fully automated Python workflow leveraging the Google Earth Engine (GEE) API for image processing and PyDrive for managing file exports and downloads from Google Drive. The goal was to extract spatially explicit flowering phenology metrics over multiple years by identifying the date on which the flowering signal—captured by NDYI—reached its maximum intensity for each pixel.

We filtered the image collections for scenes with cloud cover less than 20%. Cloud and shadow masking was performed by parsing the QA_PIXEL band, specifically masking bits 3 and 4, which correspond to clouds and cloud shadows, respectively. Each valid image was then processed to calculate NDYI and to add a separate band representing the Julian day of acquisition. NDYI, originally introduced by Sulik and Long^21^, was specifically designed to detect floral reflectance in yellow-petaled species such as rapeseed. Recent studies have demonstrated the effectiveness of NDYI in detecting rapeseed flowering dynamics, showing that the timing of maximum NDYI closely corresponds to peak flowering due to its sensitivity to the strong yellow reflectance of flowers^26,27^.

Rather than applying fixed temporal compositing windows (e.g., weekly or monthly averages), the analysis leveraged all available observations throughout the year and employed a per-pixel temporal compositing strategy using a quality mosaic approach. Specifically, the qualityMosaic(‘NDYI’) function was used to identify, for each pixel, the date at which NDYI reached its maximum value across the full time series. This approach effectively captures the peak flowering signal, as rapeseed flowering is characterized by a strong increase in yellowness reflectance. The output consists of two key layers: (1) the maximum NDYI value per pixel, representing peak floral intensity, and (2) the corresponding Julian day, representing the estimated timing of peak flowering.

### Cloud masking and data quality

Missing observations due to cloud cover or irregular satellite acquisition were handled implicitly using dense temporal stacks. Because the method relies on identifying the maximum NDYI value across all available observations, occasional gaps in the time series do not critically affect the results, provided that at least one valid observation is available during the flowering period. This approach avoids the need for interpolation or gap-filling while still capturing phenological extremes. However, the reliability of this strategy depends on temporal sampling density. Landsat 8, with a 16-day revisit interval, may be more sensitive to persistent cloud cover, potentially leading to missed peak observations in some cases. In contrast, Sentinel-2 imagery, with a 5-day revisit frequency, provides substantially denser temporal coverage and can mitigate this limitation when integrated into the workflow.

Although no formal accuracy assessment of the cloud masking procedure was conducted using independent validation data, the combination of QA-based masking, scene-level cloud filtering, and temporal compositing minimizes the impact of residual cloud contamination. Importantly, the use of a maximum NDYI compositing strategy further reduces sensitivity to remaining artifacts, as cloud-contaminated pixels typically exhibit low or noisy spectral responses and are unlikely to be selected as peak values. Nevertheless, we acknowledge that residual clouds and prolonged overcast conditions may introduce uncertainty in peak flowering estimates, particularly in years or regions with limited valid observations. This limitation is inherent to optical remote sensing and is explicitly considered in the interpretation of results.

Overall, the workflow integrates standardized preprocessing, index computation, and temporal compositing within a cloud-based platform, ensuring full reproducibility. The stepwise procedure—including data filtering, cloud masking, NDYI calculation, Julian day extraction, and maximum-value compositing—can be readily replicated using the provided code, and a schematic workflow diagram has been added to further enhance methodological transparency.

### NDYI calculation

NDYI is computed as Eq. (1):

1 $${\text{NDYI }} = {\text{ }}\left( {{\mathrm{Green}}\, - \,{\text{Blue }}} \right)/{\text{ }}\left( {{\mathrm{Green}}\, + \,{\mathrm{Blue}}} \right)$$

The index emphasizes spectral contrast in visible wavelengths, particularly where carotenoid-rich yellow petals strongly reflect green light while absorbing blue. Unlike NDVI, which captures vegetative greenness via red and NIR wavelengths, NDYI is optimized for flowering detection, not biomass or photosynthetic potential. This distinction is critical because during rapeseed’s peak flowering stage, the canopy reflectance is dominated by petal color, not chlorophyll absorption. As described by Sulik and Long^20^, flowering canopies reflect significantly more in the green region (500–600 nm) and absorb more in the blue range (400–500 nm), due to the spectral behavior of carotenoids—the pigments responsible for yellow petal coloration. Therefore, NDVI becomes less effective during flowering, as its sensitivity to red/NIR does not capture floral signals^26,27^. In contrast, NDYI accurately highlights the shift in reflectance caused by blooming flowers, making it an ideal metric for tracking the phenology of yellow-flowered crops like rapeseed.

After calculating NDYI and the Julian day for each image, we used Earth Engine’s qualityMosaic(‘NDYI’) method to produce annual composites for each pixel. This method selects the image in the time series with the highest NDYI value, interpreting it as the maximum flower cover. From this mosaic, we extracted two bands:

- Max_NDYI: the highest NDYI value for each pixel,
- Julian_Day: the calendar day on which the maximum occurred, interpreted as the peak flowering date.

## Results

**Fig. 2:**
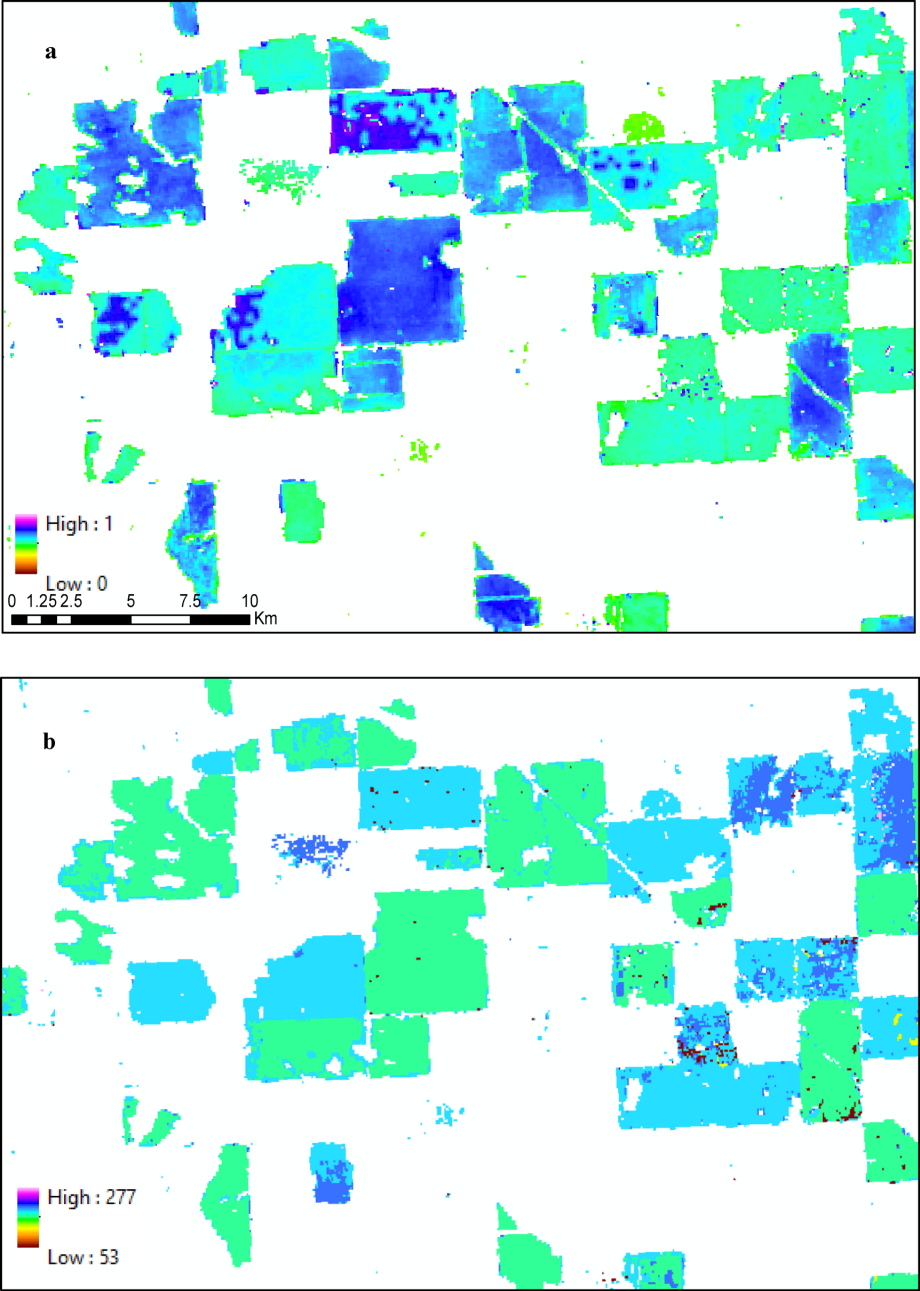
Example output from Landsat 8 for the year 2024 illustrating (**a**) the maximum of NDYI, and (**b**) the corresponding Julian dates representing the estimated peak flowering period of rapeseed.

**Fig. 3:**
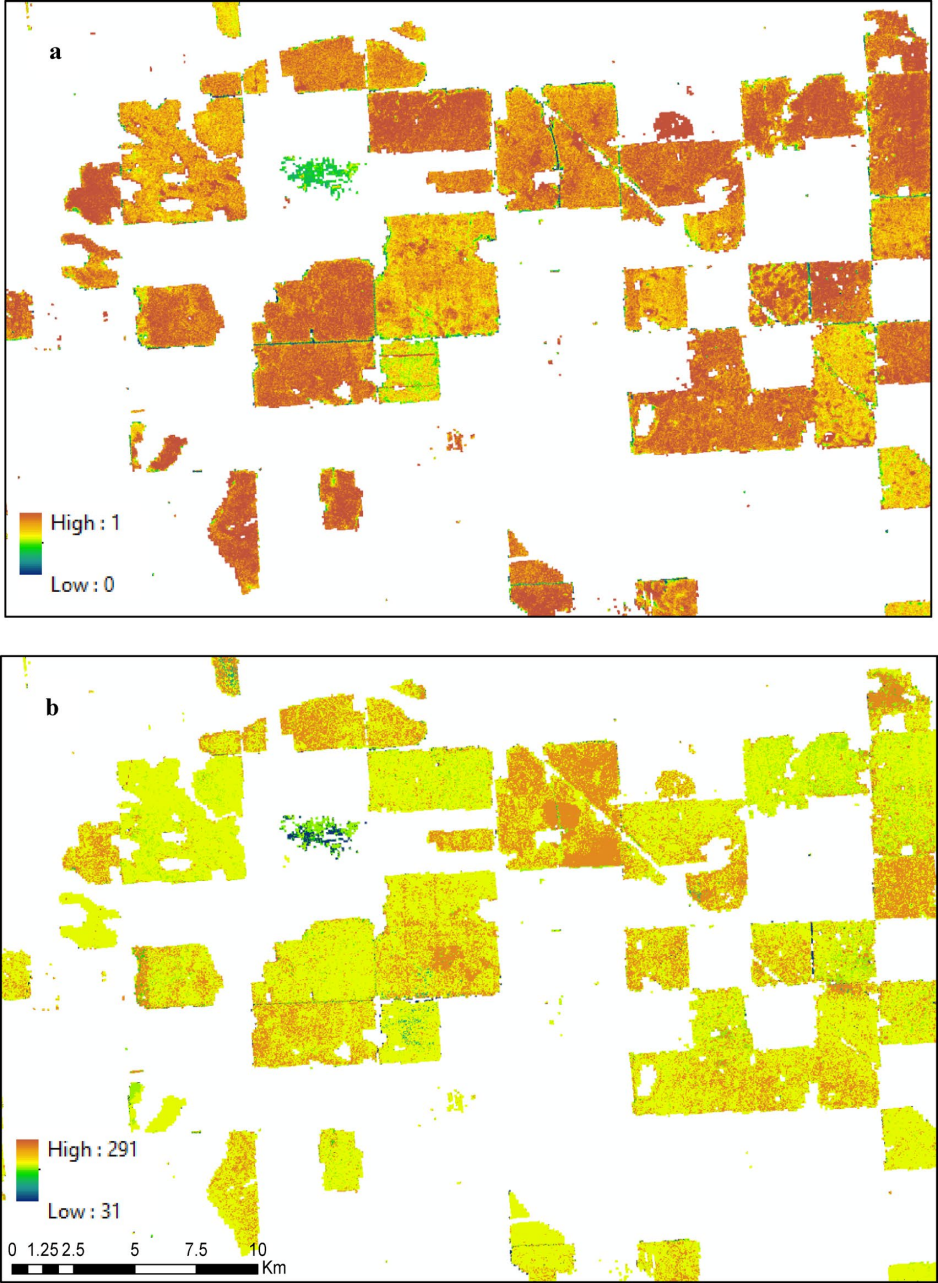
Example output from Sentinel 2 for the year 2024 illustrating (**a**) the maximum of NDYI, and (**b**) the corresponding Julian dates representing the estimated peak flowering period of rapeseed.

Figures 2 and 3 present the results for a small region in North Dakota in the year 2024, showing (a) the maximum NDYI values and (b) the detected peak flowering dates of rapeseed, derived from Landsat 8 and Sentinel-2, respectively. In both figures, spatial patterns in NDYI reveal that not all rapeseed field cells reach a value close to 1. Even within individual fields, NDYI values often vary continuously, likely reflecting differences in crop density, flower coverage, or soil background reflectance. In contrast, the maps of peak flowering dates display more homogeneity at the field level. That is, most pixels within a single field are assigned the same or similar Julian date, suggesting a consistent flowering response within fields. However, neighboring fields—even those nearby—do not always share the same flowering date, indicating variability in crop development timing across farms or management zones. Notably, Sentinel-2 data in Fig. 3 captures these spatial and temporal dynamics more clearly than Landsat 8 in Fig. 2, due to its higher spatial resolution (10 m vs. 30 m) and more frequent revisit time (5–7 days vs. 16 days for Landsat). As a result, Sentinel-2 provides finer detail in both maximum NDYI values and the distribution of flowering dates, making it particularly valuable for capturing field-level phenological variability.

### Estimated peak flowering dates

**Fig. 4:**
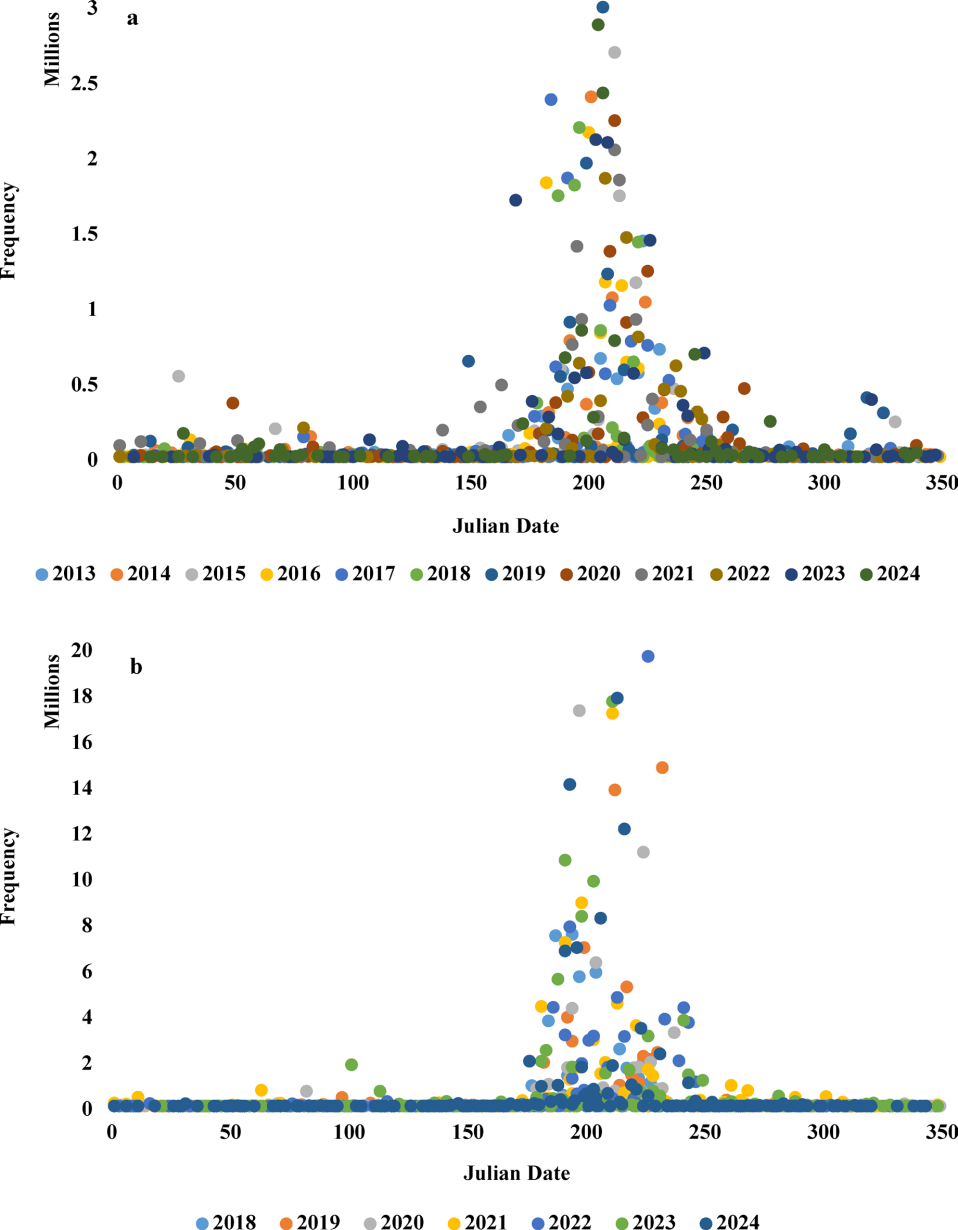
The distribution of Julian dates corresponding to the estimated peak flowering period of rapeseed in North Dakota, based on two remote sensing datasets: (**a**) Landsat 8 and (**b**) Sentinel-2 imagery.

Figure 4 illustrates the distribution of Julian dates corresponding to the estimated peak flowering period of rapeseed in North Dakota, based on two remote sensing datasets: (a) Landsat 8 imagery spanning the years 2013 to 2024, and (b) Sentinel-2 imagery covering 2018 to 2024. The analysis reveals that the majority of peak flowering dates are concentrated around Julian day 200, which corresponds approximately to July 18–19. This aligns with the known flowering phenology of spring rapeseed in North Dakota, where peak flowering typically occurs in mid-July.

Across both datasets, the distribution of flowering dates shows a standard deviation of approximately ± 30 days, reflecting some variability around the central peak. This variation is expected given the diverse environmental conditions across the state, field-level management practices, and differences in microclimates. Additionally, the presence of outlier values—dates that fall well outside the expected flowering window—can be attributed to several factors. These include cloud contamination in the satellite imagery, errors or uncertainties in the crop type classification mask (USDA CDL class 31 for rapeseed), and the inclusion of non-rapeseed vegetation within the masked NDYI outputs. Such anomalies may result in pixels showing peak NDYI values that do not coincide with the true flowering period, thus introducing minor biases in the temporal distribution.

It is also important to consider the temporal resolution of the satellite sensors, which directly influences the accuracy of phenological data detection. Landsat-8 provides an observation approximately every 16 days, while Sentinel-2 has a higher revisit frequency of around 5 to 7 days. This difference in temporal coverage affects the precision with which the peak NDYI—and hence the peak flowering date—can be determined. For instance, a flowering event occurring between two Landsat overpasses may be missed or detected with a temporal lag of several days, while Sentinel-2’s higher frequency enables more precise detection.

Given these sensor limitations and sources of uncertainty, interpreting the results at the exact day level may be misleading. Therefore, a monthly-scale interpretation is more appropriate for practical applications, especially when communicating flowering windows to agricultural stakeholders or pollination managers. In this context, both datasets consistently indicate that the month of July encompasses the majority of flowering activity for rapeseed in the region. To support and clarify the interpretation of these distributions, detailed annual statistics for each dataset—including median, mean, and range of detected peak flowering dates—are provided in Tables 1 and 2. These tables complement Fig. 2 and offer a clearer, year-by-year view of flowering phenology derived from the NDYI time series.

Table 1 summarizes the mean, median, and standard deviation of the estimated Julian dates for peak rapeseed flowering in North Dakota, based on Landsat 8 imagery from 2013 to 2024. The results reveal that, across most years, the estimated mean and median flowering dates fall between Julian days 196 and 221, corresponding to the first three weeks of July. Despite general consistency, there is noticeable inter-annual variability. For example, in 2013 and 2021, the median flowering date occurred relatively late (Julian day 221 and 211, respectively), while in 2017 and 2018, the median dates were earlier, around Julian days 191–196. This variability may reflect differences in weather patterns, planting dates, or crop management across years. The standard deviation values, ranging from 26 to 58 days, indicate the level of within-year spatial variability in flowering times across rapeseed fields. Years like 2015 and 2020 show higher variability (StdDev = 58 and 48, respectively), suggesting greater heterogeneity in flowering phenology during those seasons, potentially due to patchy field conditions, uneven flowering, or limitations in Landsat’s 16-day revisit interval for capturing phenological peaks with precision.

Table 2 presents the mean, median, and standard deviation of the estimated Julian dates for peak rapeseed flowering, based on Sentinel-2 imagery from 2018 to 2024. Compared to the Landsat-based estimates, the Sentinel-2 data—with its higher temporal (5–7 days) and spatial (10 m) resolution—offers finer detail and greater temporal accuracy in capturing flowering peaks. Across the seven years analyzed, the mean flowering dates generally range from Julian day 203 to 230, corresponding to the early to late July period. Similarly, median dates fall between Julian days 202 and 216, confirming that most peak flowering events are concentrated in mid-July, consistent with known rapeseed phenology in North Dakota. The standard deviation values are generally lower than those from Landsat, especially in years like 2019, 2020, 2022, and 2024, where the StdDev ranges from 16 to 22 days. This suggests a more precise temporal detection of flowering peaks and less intra-annual variability at the pixel level, likely due to Sentinel-2’s more frequent observations. However, 2018 shows an unusually high standard deviation (63 days), possibly due to atmospheric interference, early-season anomalies, or cloud-related gaps in imagery coverage.

**Table 1:** Mean, median, and standard deviation of the estimated Julian dates of peak rapeseed flowering, derived from Landsat 8 data for the period 2013–2024.

	2013	2014	2015	2016	2017	2018	2019	2020	2021	2022	2023	2024
Mean	211	203	197	198	203	199	205	212	196	207	213	201
Median	221	201	211	205	191	196	206	211	211	207	208	204
StdDev	26	37	58	29	35	30	46	48	41	27	44	35

**Table 2:** Mean, median, and standard deviation of the estimated Julian dates of peak rapeseed flowering, derived from Sentinel 2 data for the period 2018–2024.

	2018	2019	2020	2021	2022	2023	2024
Mean	230	213	207	204	214	203	205
Median	202	212	204	208	216	203	206
StdDev	63	21	22	33	21	27	16

## Discussion

This study aimed to estimate the peak flowering period of rapeseed across North Dakota using time series of satellite-derived NDYI from both Landsat 8 and Sentinel-2. The core goal of our analysis was to map the timing of maximum NDYI—used as a proxy for peak flowering—over multiple years, providing insights into both spatial and temporal patterns. As expected, most of the the detected peak flowering dates fell around Julian day 200, which corresponds to mid-July in North Dakota, aligning well with known phenological stages of spring rapeseed in this region. However, substantial variation was observed within and between years, attributable to environmental heterogeneity, crop management differences, and sensor-specific limitations.

The mean Julian dates for Landsat-derived flowering ranged between 196 and 213, while Sentinel-derived estimates ranged from 203 to 230. On average, Sentinel-2 detected slightly later flowering dates compared to Landsat. For instance, in 2018, the mean date from Landsat was 199, whereas Sentinel reported 230—a considerable difference of 31 days, likely due to either missing early-season data in Sentinel (due to cloud interference) or better detection of peak yellowness occurring slightly later in that season. In other years, however, the differences were smaller (e.g., 2021: Landsat mean = 196; Sentinel mean = 204), suggesting reasonable agreement.

The median values also show divergence, though with some overlap. Sentinel median Julian dates ranged from 202 to 216, while Landsat medians ranged from 191 to 221. In several years, Landsat produced earlier medians, such as in 2017 (median = 191), compared to Sentinel (not available that year). In overlapping years, Sentinel medians were generally closer to the expected flowering window of mid-July, providing more phenologically plausible estimates. This indicates that Sentinel’s finer temporal resolution likely allowed it to capture the actual flowering event more precisely, whereas Landsat’s lower revisit frequency may have led to capturing either pre-peak or post-peak values in some cases.

The most notable differences between the two sensors emerge in the standard deviation (SD) of flowering dates. Landsat’s SD ranged from 26 to 58 days, while Sentinel’s was consistently lower, between 16 and 33 days, except for 2018 (SD = 63), which likely suffered from data quality issues. This contrast indicates that Sentinel-2 offered more stable and consistent flowering date estimates, with less within-year spatial variability. This can be attributed to Sentinel’s higher revisit rate (5–7 days) and finer spatial resolution (10 m), which help reduce uncertainty due to temporal sampling gaps and mixed-pixel effects.

The satellite-derived flowering dates obtained in this study are broadly consistent with reported phenological timings from the literature, while also highlighting some sensor-specific differences. Our results indicate that the mean Julian dates of peak flowering ranged from approximately 196 to 213 for Landsat and 203 to 230 for Sentinel-2, corresponding roughly to mid-July through mid-August. These estimates align well with previous studies from climatically comparable regions. For example, in Manitoba, Canada, rapeseed flowering typically begins in the last week of June to early July, followed by pod development in mid-July and senescence from late July to mid-August^28^. Similarly, in North Dakota, anthesis is generally reported around mid-July^21^, which corresponds closely to the central tendency of both Landsat- and Sentinel-derived estimates in our analysis. Additional field-based observations from Saskatchewan indicate that flowering can extend from late June (e.g., 27 June, BBCH 61) to early August (e.g., 9 August, BBCH 67)^1^, further supporting the temporal window identified in this study.

Despite this overall agreement, some differences are evident. Sentinel-2 tends to estimate slightly later peak flowering dates compared to Landsat, in some cases by several weeks (e.g., 2018). These later estimates, however, often fall closer to the expected peak flowering window (mid-July), suggesting that Sentinel-2 may more accurately capture the true flowering maximum. This is likely due to its higher temporal resolution (5–7 days), which increases the probability of acquiring cloud-free observations during the relatively short flowering period. In contrast, Landsat’s 16-day revisit interval can result in missed peak conditions, leading to earlier or more variable estimates, as reflected in its wider range and higher standard deviation. Nevertheless, both sensors consistently capture flowering within the broader phenological window reported in the literature (late June to early August), indicating that the NDYI-based approach provides realistic and ecologically meaningful estimates of flowering dynamics. Overall, strong agreement with independent agronomic and field-based studies supports the validity of the proposed method, while also demonstrating the advantage of higher temporal resolution data for phenological applications.

Our results also demonstrated that NDYI, a flower-sensitive spectral index, is a reliable and effective indicator for detecting the peak flowering period of rapeseed. Previous work has shown strong correlations between NDYI and flower density, further validating its application in flowering phenology^29^. Also, our methodology holds practical significance for agronomic scheduling, yield prediction, and pollination service management, particularly as rapeseed serves as a major source of nectar and pollen for pollinators during its flowering phase^6^. Accurate detection of peak bloom supports the timely deployment of beehives and enhances landscape-scale assessments of floral resource availability. However, this approach is not without limitations. NDYI relies on optical remote sensing, which is inherently affected by cloud cover, atmospheric conditions, and solar illumination geometry, especially during rapid phenological transitions^30^. These factors can lead to gaps or inaccuracies in the time series, particularly in systems with limited temporal resolution such as Landsat. Although Sentinel-2 mitigates some of these issues through higher spatial and temporal coverage, optical sensors remain constrained by cloud interference, which can obscure critical observations.

To overcome this, Synthetic Aperture Radar (SAR) imagery offers a promising complementary approach. SAR sensors, such as those on Sentinel-1, operate independently of daylight and cloud cover, making them highly valuable for consistent monitoring throughout the growing season^31^. While SAR does not directly detect floral color, changes in canopy structure and moisture associated with flowering can influence backscatter signals, enabling indirect phenological assessment^17,32,22^. Integrating optical indices like NDYI with SAR-based phenology metrics could provide a more robust and all-weather system for flowering detection in future studies^33,5^.

## Conclusion

This study demonstrated the effectiveness of NDYI in detecting the peak flowering period of rapeseed across North Dakota using multi-year satellite imagery. Sentinel-2 outperformed Landsat 8 in temporal precision and consistency, owing to its higher spatial and temporal resolution. Most flowering events were consistently detected around mid-July (Julian day ~ 200), aligning with known rapeseed phenology. NDYI proved to be a reliable indicator of flowering intensity, offering valuable insights for crop management, pollination planning, and floral resource monitoring. Despite the strengths of optical remote sensing, cloud-related limitations remain, highlighting the potential value of integrating SAR data for more resilient, all-weather phenological monitoring systems.

## Data Availability

Python codes for calculating NDYI from both Landsat and Sentinel-2 imagery are available. at: https://github.com/ehsanrahimi666/North-Dakota.git.
